# Micronutrient Fortified Condiments and Noodles to Reduce Anemia in Children and Adults—A Literature Review and Meta-Analysis

**DOI:** 10.3390/nu8020088

**Published:** 2016-02-15

**Authors:** Sascha Hess, Linda Tecklenburg, Klaus Eichler

**Affiliations:** Winterthur Institute of Health Economics, Zurich University of Applied Sciences, Winterthur 8401, Switzerland; tecklin@web.de (T.L.); klaus.eichler@zhaw.ch (E.K.)

**Keywords:** micronutrients, iron, fortification, condiments, noodles, meta-analysis, review

## Abstract

Micronutrient deficiencies impose a considerable burden of disease on many middle and low income countries. Several strategies have been shown to be effective in improving micronutrient deficiencies. However, the impact of fortified condiments as well as fortified noodles is less well documented. We aimed to investigate existing evidence on the impact of micronutrient fortified condiments and noodles on hemoglobin, anemia, and functional outcomes in children and adults (age: 5 to 50 years). We conducted a literature review in electronic databases. In addition, we screened the homepages of relevant organizations and journals. We included randomized controlled trials (RCT). Of 1046 retrieved studies, 14 RCT provided data for the meta-analysis. Micronutrient fortification of condiments and noodles increased hemoglobin concentrations by 0.74 g/dL (95%-confidence intervals (95%-CI): 0.56 to 0.93; 12 studies) and 0.3 g/dL (95%-CI: 0.12 to 0.48; 1 study), respectively. Micronutrient fortification also led to a reduced risk of having anemia (risk ratio 0.59 (95%-CI 0.44 to 0.80)). Ferritin concentrations increased with fortified condiments. Functional outcomes were rarely assessed and showed mixed results. The use of micronutrient fortified condiments can be a strategy to reduce anemia in children and adults due to micronutrient deficiencies. The effect of fortified noodles seems to be smaller.

## 1. Introduction

Micronutrient (MN) deficiencies impose a considerable burden of disease on many middle- and low-income countries, resulting for example in reduced growth, high anemia prevalence, or increased infection rates [1]. Several strategies, proposed in recommendations and guidelines, have been shown to be effective in preventing or reducing MN deficiencies in different target populations and with different carriers for MN [2,3,4]: Examples include food-based approaches (such as spreads to increase energy-density and MN content of food; MN powders for home fortification with sprinkles; fortified condiments) and MN supplementation (such as vitamin A capsules administered at defined intervals). Fortification of staple food (such as fortified salt, noodles, flour or oil) is also widely used to resolve MN deficiencies in general populations.

Distribution of fortified food via government programs does not always work well due to logistical problems or inappropriate priority setting. Thus, commercially distributed fortified foods that fit local nutrition habits may be an additional option to improve nutritional status in developing countries. Fortified condiments (widely consumed in Africa and Asia) and fortified noodles (specifically consumed in Eastern-Asia) are two such centrally processed nutrition types which are already on the market, even in low-income countries.

Research on fortification of milk products for infants and children has shown that multi-micronutrient fortification can considerably reduce anemia rates [5,6]. Several systematic reviews exist, that have assessed the impact of fortification for different target populations and different carriers for MN [2,7,8,9,10,11]. However, the effects of fortified condiments as well as fortified noodles are less well documented.

The aim of this study was to assess the impact of micronutrient fortified condiments and noodles on hemoglobin, anemia, and functional outcomes in children and adults from 5 to 50 years of age.

## 2. Experimental Section

We conducted a literature review and meta-analysis of RCT to address the research question as formulated above.

The literature review took into account critical methodological issues as described in the Center for Reviews and Dissemination (CRD) guidelines for undertaking systematic reviews [12]. The reporting is based on the current PRISMA statement [13]. A review protocol was developed in advance (see online supplementary material).

### 2.1. Search Strategy

We performed a literature search in MEDLINE and the COCHRANE-Library (from inception to December 2013). The details of the Pubmed search strategy are listed in Table 1. We performed a similar search in the Cochrane Library with adapted Cochrane search terms. Included articles were screened for new references and systematic reviews were used to detect other potentially relevant primary studies. Additionally, we screened homepages of relevant organizations (such as WHO, United Nations (World Food Program, United Nations Children’s Fund (UNICEF), Millenium Development Goals), The World Bank, Pakistan National Nutrition Survey, International Clinical Epidemiology Network, Global Alliance for Improved Nutrition (GAIN), Micronutrient Initiative, and Bill & Melinda Gates Foundation). We also performed searches in the Lancet journal, because of the focus on developing countries, and in Google scholar. We contacted a manufacturer (Nestlé), as a producer of centrally processed food, for additional literature [12]. We also screened an existing literature database, which has been compiled during a larger project about fortification of milk and cereal products for micronutrient deficient populations. No restriction on publication date or language was imposed. Analog search terms as for electronic databases were used for the searches in the Lancet journal (using the “advanced search” tool), Google scholar and the homepages of relevant organizations.

**Table 1:** Pubmed electronic search strategy.

Step	Search Pubmed
1	Fortif *
2	Condiments [MesH] OR Seasoned OR Seasoning OR Bouillon * OR Sprinkle * OR Soy sauce *
3	OR Fish sauce* OR Powder */NOT milk powder * OR Noodle *
4	1 AND 2 Fortified salt *

### 2.2. Inclusion/Exclusion Criteria

To answer our research question, we defined the following inclusion criteria:

**Population**: Children and adults from 5 to 50 years. **Intervention**: Micronutrient fortified condiments or noodles products. Fortified condiments include: condiments, salt, seasonings, soy sauce, fish sauce, bouillon, sprinkle, powder. Micronutrients for fortification include: iron, vitamins, zinc, iodine, folate, calcium, phosphorus, magnesium, selenium. **Control intervention**: Non-fortified condiments and noodles. **Outcome**: Serum markers with direct health impact (such as hemoglobin; anemia rate), functional status, quality of life, morbidity (as measured with physical or mental health measures), or mortality. We also included socioeconomic outcomes, productivity, or acceptability of fortified products. **Design**: Randomized controlled trials, RCT.

We excluded studies with children under 5 years and adults over 50 years. Nutritional intervention solely based on supplementation, salt fortified only with iodine, or interventions to test the bioavailability of micronutrients were excluded. Fortified staples, oils, or herbs were also not included. Change in surrogate parameters (e.g., MN blood concentrations) was not an included outcome. Surrogate parameters can be relevant from a nutrition science perspective, but they do not by themselves facilitate a comparison of the direct health impact of the intervention.

### 2.3. Study Selection and Data Extraction

In advance, we conducted training sessions to increase consistency between reviewers. Two reviewers screened titles and abstracts for relevance. Unclear cases were discussed between reviewers, and if necessary, with a third reviewer. Disagreements were resolved by consensus.

Potentially relevant studies were assessed by full text according to the pre-specified inclusion and exclusion criteria. A data extraction form was developed using Microsoft Excel, pilot tested on a small selection of studies and adjusted as necessary. Data were extracted by one reviewer and checked independently by a second reviewer.

The following information was extracted: study identifying items (such as author, year, location, and setting), population details (such as number of participants, age, sex, exclusion criteria), intervention details (such as carrier of MN, type of fortification, and concentration), results (such as numerical data for effectiveness/Hb and Ferritin, adverse events). If several papers reported results from the same population, each population was included only once for analysis.

To assess risk of bias of the included studies, quality assessment forms were developed on Microsoft Excel using current guidelines for the conduct of interventional studies [12]. We included the following domains: randomization (generation of random sequence; allocation concealment); blinding of participants; incomplete outcome data; and selective outcome reporting. According to pre-specified criteria for risk-of-bias assessment, data were extracted for each included RCT. For example, outcome data were deemed complete, if follow-up data were provided for at least 80% of participants or missing outcome data had been imputed (for details of other criteria, please see study protocol). We used the following categories: YES (criterion fulfilled), NO (criterion not fulfilled) and “?” (unclear, not enough information given). Risk-of-bias assessment was carried out by one reviewer. Unclear cases were discussed with a second reviewer. Disagreements were resolved by consensus.

### 2.4. Analysis

We performed an analysis with statistical pooling for continuous variables like hemoglobin changes (forest plots of weighted mean differences and 95%-confidence intervals (CI)). If means and standard deviations of changes were not reported, we calculated change as the difference between baseline and final values for the intervention and control group and applied the SD of final values [7]. For anemia rates, we calculated risk ratios and 95%-CI. We calculated the heterogeneity between the analyzed studies with *I*^2^, which is the percentage of the variation in the estimated effects due to heterogeneity rather than by chance [14]. *I*^2^ lies between 0% and 100%. A rough guide to interpretation is as follows: Heterogeneity of 0% to 40% might not be important; a range of 30% to 60% may represent moderate, 50% to 90% substantial, and 75% to 100% considerable heterogeneity. We tested the robustness of results by calculating weighted mean differences for hemoglobin concentrations at the end of follow-up.

Furthermore, we divided our studies into reasonable subgroups, as pre-specified in our protocol, and performed subgroup analyses depending on the data available. Domains for subgroups comprised fortification strategy (single MN strategy *vs.* dual/multi MN strategy), region (Asia *vs*. Africa), food carrier (condiments *vs*. noodles), type of iron salt (NaFeEDTA *vs*. other salts) and risk of bias (low *vs.* intermediate/high). Studies with low risk of bias were defined as fulfilling at least four out of five quality domains (“YES”). An ex-post subgroup analysis compared targeted populations (children or adolescents *vs.* other populations (mostly women in childbearing age; sometimes entire communities)).

Finally, we performed a meta-regression analysis weighted for the inverse of the variance of the outcome to assess the influence of single parameters on hemoglobin change [14]. Such parameters were hemoglobin concentrations before intervention, follow-up completeness, and length of follow-up.

Significance *p*-values of <0.05 were used. Analyses were carried out with STATA SE 12.1 software package (Stata-Corp. 2011. Stata Statistical Software, College Station, TX, USA).

## 3. Results

### 3.1. Description of Included Studies

**Figure 1:**
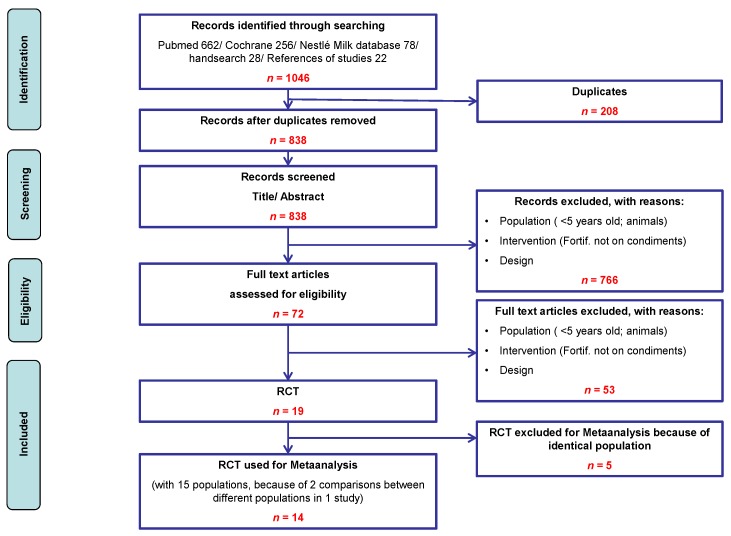
Flow-chart of studies in the review process.

Our searches in the different sources retrieved 1046 potentially relevant studies (Figure 1). 19 RCT fulfilled inclusion criteria [15,16,17,18,19,20,21,22,23,24,25,26,27,28,29,30,31,32,33]. Five studies had to be excluded to avoid double-counting of the same populations. Of the remaining 14 RCT, 15 comparisons could be used for the meta-analysis (Table 2). 11 of 14 studies were conducted in Asia (India [25,26,27,28], Vietnam [16,17,18], China [32,33], Thailand [30], Cambodia [15]) and three in Africa (Morocco [24], Ghana [21], South Africa [20]). 

The carrier used for micronutrients was salt six times [21,24,25,26,27,28], fish sauce three times [15,16,17], soy sauce twice [32,33], and seasoning [30], noodles [18], and masala powder once [20]. Only in two trials was multi-micronutrient (MMN) fortification [28,30] used, e.g. additional fortification with vitamin A, zinc, and folate. In the other 12 studies [15,16,17,18,20,21,24,25,26,27,31,33], iron was the net difference in MN exposition between intervention and control groups. For example, some studies used a dual MN strategy with iron and iodine in the intervention group and mono-iodized salt in the control group, while other studies used a single MN strategy with iron in the intervention group and non-fortified food in the control group.

**Table 2:** Details of included RCT studies for micronutrient fortified condiments and noodles.

Author; Year	Population	Intervention	Control	Outcome Parameters (Including Surrogate Parameters)
Andersson; 2008 [25] Design: RCT	Country: India Age in years (mean (range)): 12 (5 to15) Males in %: 52 Exclusion criteria : Hb < 8 g/dL	*n* = 130 Dual fortified salt providing iron and iodine (potassium iodate (KIO3; 30 µg/g salt) and MGFePP (2 mg/g salt)) MN strategy: dual MN strategy	*n* = 131 (Mono) iodized salt providing potassium iodate (KIO3; 30 µg/g salt)	After 0.8 years: Anemia rate, Hb, ferritin, transferrin, body iron, zinc protoporphyrin, acceptability
Asibey-Berko; 2007 [21] Design: RCT	Country: Ghana Age in years (mean (range)): 29 Males in %: 0 Exclusion criteria : pregnant women, unwilling to take fortified salt, Hb < 100 g/L	*n* = 65 Dual fortified salt providing iron and iodine (potassium iodide (50 mg/kg salt) and ferrous furamate (1 g/kg salt)) MN strategy: dual MN strategy	*n* = 58 (Mono) iodized salt providing potassium iodate (50 mg/kg salt) and placebo	After 0.7 years: Anemia rate, urinary iodine
Ballot; 1989 [20] Design: cluster RCT (264 clusters: families)	Country: South Africa Age in years (mean (range)): no info Males in %: 45.6 Exclusion criteria : age < 10 year, Hb ≤ 90 g/L	*n* = 298 Fortified masala powder providing iron (NaFeEDTA (25 µg/g masala)) MN strategy: single MN strategy	*n* = 300 Non fortified masala powder	After 2 years: Hb, ferritin, transferrin saturation, body iron
Chen; 2005 [33] Design: cluster RCT (9 clusters: villages)	Country: China Age in years (mean (range)): no info (3 to 55+) Males in %: no info Exclusion criteria : no info	*n* = 2020 Fortified soy sauce providing iron (NaFeEDTA (29.6 mg/100 mL soy sauce )) MN strategy: single MN strategy	*n* = 2007 Non fortified soy sauce	After 1.5 years: Hb, ferritin, serum retinol, W/H, food consumption, anemia rate
**Author; year**	**Population**	**Intervention**	**Control**	**Outcome parameters (including surrogate parameters)**
Huo; 2002 [32] Design: RCT	Country: China Age in years (range): 11 to 17 Males in %: 51 Exclusion criteria : no anemia	*n* = 77 Fortified soy sauce providing iron (high-NaFeEDTA (4 mg Fe/mL soy sauce)) MN strategy: single MN strategy	*n* = 81 Non fortified soy sauce	After 0.2 years: Anemia rate, Hb, ferritin, transferrin, iron, free erythrocyte protoporphyrin, total iron binding capapility
Le; 2007 [18] Design: RCT	Country: Vietnam Age in years (mean (range)): 7.3 Males in %: IG:51.2 CG:48.8 Exclusion criteria : Hb < 70g /L	*n* = 86 Fortified noodles providing iron (NaFeEDTA (10.7 mg/52 g noodles)) MN strategy: single MN strategy	*n* = 82 Non fortified noodles with placebo	After 0.5 years: Anemia rate, Hb, ferritin, serum transferrin rezeptor, body iron
Le; 2007 [18] Design: RCT	Country: Vietnam Age in years (mean (range)): 7.3 Males in %:48.7 Exclusion criteria : Hb < 70 g/L	*n* = 79 Fortified noodles providing iron (NaFeEDTA (10.7 mg/52 g noodles)) with mebendazole MN strategy: single MN strategy	*n* = 79 Non fortified noodles with mebendazole	After 0.5 years: Anemia rate, Hb, ferritin, serum transferrin rezeptor, body iron
Longfils; 2008 [15] Design: RCT	Country: Cambodia Age in years (mean (range)): 13.6 (6 to 21) Males in %: 40 Exclusion criteria : acute malnutrition below 80% weight or height score, Hb < 70 g/L, chronic diseases, iron supplementation, lack of parental approval	*n* = 46 Fortified fish sauce providing iron (NaFe-EDTA (1 mg Fe/mL fish sauce)) MN strategy: single MN strategy	*n* = 44 Non fortified fish sauce (endogenous iron content of 86 mg Fe/L)	After 0.4 years: Hb, ferritin, CRP, W/H, BMI, infections (vomiting, diarrhea, acute respiratory infection)
Rajagopalan; 2000 [26] Design: cluster RCT (20 clusters: housing areas)	Country: India Age in years (mean (range)): no info (18 to 45) Males in %: 39 Exclusion criteria : casual workers	*n* = 385 Dual fortified salt providing iron and iodine (no info about MN dosage) MN strategy: dual MN strategy	*n* = 408 (Mono) iodized salt	After 1 year: Hb, productivity in tea leaf picking
Thuy; 2003 [17] Design: RCT	Country: Vietnam Age in years (mean (range)): 34 (19 to 49) Males in %: 0 Exclusion criteria : women with gastrointestinal or metabolic disorders, pregnant women	*n* = 64 Fortified fish sauce providing iron (NaFeEDTA (1 mg Fe/mL fish sauce)) MN strategy: single MN strategy	*n* = 72 Non fortified fish sauce	After 0.5 years: Anemia rate, Hb, ferritin, transferrin
Thuy; 2005 [16] Design: cluster RCT (21 clusters: villages)	Country: Vietnam Age in years (mean (range)): 32 (16 to 49) Males in %: 0 Exclusion criteria : no info	*n* = 199 Fortified fish sauce providing iron (NaFeEDTA (9 mmol Fe/L fish sauce)) MN strategy: single MN strategy	*n* = 190 Non fortified fish sauce	After 1.5 years: Anemia rate, Hb, ferritin, CRP, serum retinol, intestinal parasites (eggs counts)
Vinodkumar; 2007 [27] Design: cluster RCT (7 clusters: communities)	Country: India Age in years (mean (range)): no info Males in %: 39 Exclusion criteria : no info	*n* = 393 Dual fortified salt providing iron and iodine (potassium iodate (40 µg/g salt), ferrous sulfate monohydrate (1 mg/g salt)) MN strategy: dual MN strategy	*n* = 436 (Mono) iodized salt providing potassium iodate	After 1 year: Hb, urinary iodine
Vinodkumar; 2009 [28] Design: cluster RCT (6 clusters: schools)	Country: India Age in years (mean (range)): 12.3 (5 to 18) Males in %: no info Exclusion criteria : schools where children go home more than once a year	*n* = 213 Multimicronutrient fortified salt providing iron, zinc, vitamin A, other vitamins, folate, other MN (10 g salt contained the RDA of MN. Iron 1 mg/g salt.) MN strategy: Multimicronutrient strategy	*n* = 18 (Mono) iodized salt providing potassium iodate	After 0.75 years: Anemia rate, Hb, ferritin, serum transferrin rezeptor, body iron stores, CRP, alkaline granulocytes phosphatase, serum Vit.A, serum Vit. B12, serum folic acid, serum zinc, angular stomatitis, memory Test
Winichagoon; 2006 [30] Design: cluster RCT (10 cluster: schools)	Country: Thailand Age in years (mean (range)): 9.2 (5.5 to 13.4) Males in %: 49.5 Exclusion criteria : acute or chronic illnesses, Hb < 80 g/L	*n* = 278 Multimicronutrient fortified seasoning providing iron, zinc, iodine, vitamin A (per serving (lunch): 5 mg H-reduced elemental iron, 270 µg palmitate, 50 µg potassium idodine, 5 mg zinc sulfate) MN strategy: Multimicronutrient strategy	*n* = 277 Non fortified seasoning powder	After 0.6 years: Anemia rate, Hb, ferritin, urinary iodine, serum retinol, mean corpuscular volume, W/H, BMI, arm circumfences infection status, short-term learning, memory, attention span
Zimmermann; 2004 [24] Design: cluster RCT (clusters: households; no information about number of clusters)	Country: Morocco Age in years (mean (range)): 10.7 (6 to 15) Males in %: 53 Exclusion criteria : no info	*n* = 75 Dual fortified salt providing iron and iodine (potassium iodate (25 µg iodine/g salt) and FePP (2 mg Fe/g salt)) MN strategy: dual MN strategy	*n* = 83 (Mono) iodized salt providing potassium iodate (25 µg iodine/g salt)	After 0.8 years: Anemia rate, Hb, ferritin, transferrin, CRP, zinc, urinary iodine, thyroid volume, goiter, color, taste acceptability, satisfaction with salt, sensory changes

**Abbreviations**: BMI: body mass index; CRP: C-reactive protein; Hb: hemoglobin; MN: micronutrient; n = number of participants; W/H: body weight and standing height; RCT: randomized controlled trial; RDA: recommended daily allowance.

The 14 studies with 15 comparisons (one study provided two comparisons between four different groups [34]) comprised 8845 people with a mean age between 7.3 and 34 years. Follow-up periods were mostly under one year (mean follow-up: 0.88 years; range: 2.4 months to 2 years). Most participants lived in a rural region. In two trials, participants were women of childbearing age. The most common setting for recruitment was the school setting (nine trials). The populations included in the primary studies were representative of the target population for fortified food, *i.e.*, MN deficiencies were common. However, persons with severe disease (e.g. anemia with Hb < 8g/L), were often excluded from the RCTs. Adherence to intervention was not reported.

### 3.2. Effects on Hemoglobin Concentrations

**Figure 2:**
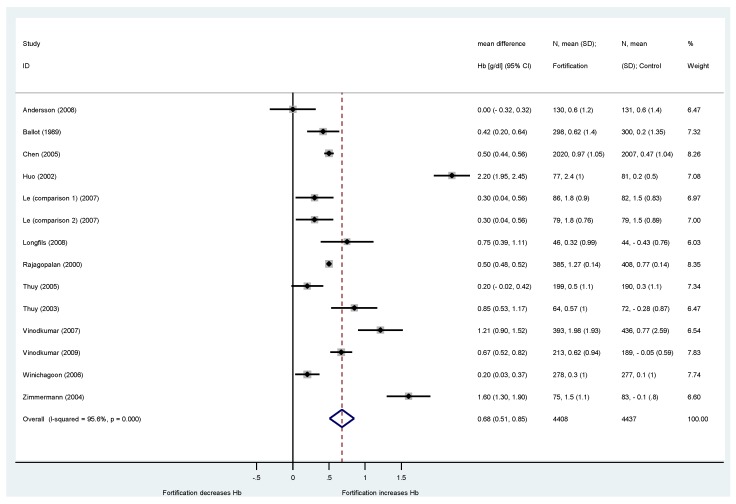
Effect of fortified condiments and noodles on hemoglobin (Hb) concentrations compared to non-fortified condiments or noodles. Included are 13 studies with 14 comparisons. Results are provided as weighted mean difference in hemoglobin (WMD: g/dL with 95%-CI) between intervention and control group.

Hemoglobin concentrations in blood was the most frequently reported outcome parameter. Median baseline hemoglobin concentrations were 11.8 g/dL (IQR: 11.1 to 12.5) for intervention groups and 12.0 g/dL (IQR: 11.0 to 12.4) for control groups. Micronutrient fortification of condiments and noodles (mostly iron as the only difference between compared groups) increased hemoglobin concentrations by 0.68 g/dL (95%-CI: 0.51 to 0.85; *I*^2^ = 96%; 13 RCT with 14 comparisons and 8845 participants) compared to control groups (Figure 2).

Comparison of pre-specified subgroups in different domains showed no statistically different effect on hemoglobin concentrations for fortification strategy (single MN strategy *vs.* dual/multi MN strategy) and region. Hemoglobin concentrations showed a more pronounced increase in the 11 studies with high risk of bias (0.78 g/dL; 95%-CI: 0.47 to 1.08) compared to the two studies (three comparisons) with low risk of bias (0.41 g/dL; 95%-CI: 0.26 to 0.57), but again, the difference was not statistically different. Condiments showed a higher impact on hemoglobin change (increase of 0.74 g/dL; 95%-CI: 0.56 to 0.93) than noodles (increase of 0.3 g/dL; 95%-CI: 0.12 to 0.48), but data for noodles were from one single study. Finally, different types of iron preparations showed no differences in rise of hemoglobin concentrations (NaFeEDTA: 0.69 g/dL *vs.* 0.68 g/dL for other preparations). Also our ex-post subgroup analysis showed no relevant difference between children/adolescents and other targeted populations.

### 3.3. Effects on Anemia Prevalence

For the definition of anemia, most studies relied on the WHO definition [35] and used thresholds between 11 g/dL and 13 g/dL, depending on age and gender of the investigated population. The median of anemia rates at baseline was 46% (IQR: 26% to 95%). Six studies reported iron deficiency anemia rates based on ferritin concentrations (median: 55% (IQR: 38% to 77%) in this subgroup). Again, in most of the studies, iron fortification was the only difference between intervention and control groups.

**Figure 3:**
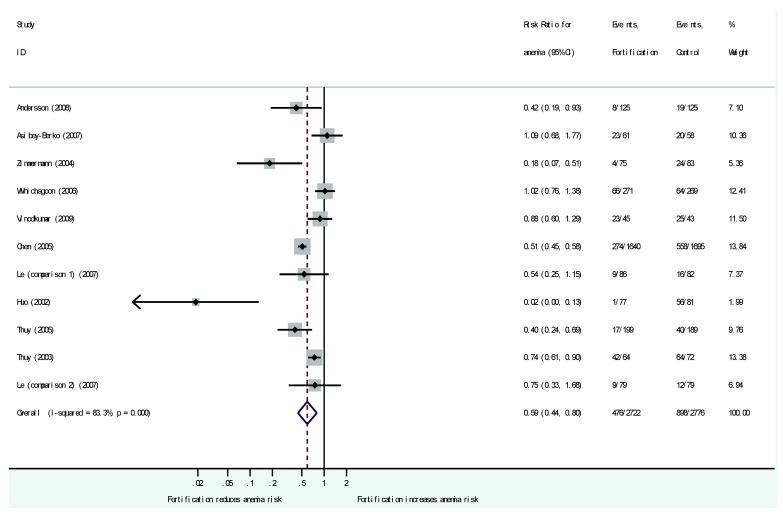
Effect of fortified condiments and noodles on anemia rate compared to non- fortified condiments or noodles. Included are 10 studies with 11 comparisons. Results are provided as risk ratio (RR, 95%-CI) of suffering from anemia in the intervention group compared to the control group.

The risk of having anemia in the intervention groups compared to control groups was 0.59 (95%-CI: 0.44 to 0.80; *I*^2^ = 83%) in 10 RCT (11 comparisons; Figure 3). Similar anemia rates emerged from the comparison of studies with high and low risk of bias (high risk: 0.58; 95%-CI: 0.42 to 0.81, low risk: 0.63; 95%-CI: 0.36 to 1.1). Also in the five other subgroup domains (fortification strategy; region; food carrier; type of iron salt; targeted populations) no significant differences emerged for anemia rates.

### 3.4. Effect on Ferritin Concentrations

Median baseline ferritin concentrations were 13.5 micro-g/L (IQR: 8.0 to 20.5) for intervention groups and 13.4 micro-g/L (IQR: 9.5 to 18.5) for control groups. The meta-analysis of ferritin concentrations is based on three studies with mean values [16,32,33]. The mean ferritin increase with fortified condiments (fish or soy sauce) was 1.94 micro-g/L (95%-CI: 0.9 to 3.0; *I*^2^ = 86%). Five trials reported medians [15,17,20,25,28]. The median of medians after intervention was 19.7 micro-g/L (IQR: 19.6 to 30.9) in the intervention groups and 12.2 micro-g/L (IQR: 11.6 to 14.6) in the control groups.

### 3.5. Other Reported Effects

Outcomes other than hemoglobin concentrations, anemia rates, or ferritin concentrations are rarely assessed in the primary studies. Five studies additionally reported morbidity outcomes, two studies assessed cognition and one trial described productivity outcome. Among these few studies, no consistent pattern of relevant effects of micronutrient fortification of condiments or noodles emerged on outcomes like infections and anthropometric parameters. Consumer acceptability of salt was not influenced by fortification.

One RCT from India [28] compared multi-micronutrient salt with iodized salt in schoolchildren and found a significantly higher memory score (ability to memorize words in the same sequence as played by audiotape before) in the intervention group than in the control group.

One randomized trial from Thailand [29] looked at the cognitive function (short term learning, memory, attention span, visual recall) of primary-school children with or without iron-fortified salt. After 31 weeks, they found a significant intervention effect of iron-fortified salt only on visual recall.

Another trial from India [26] reported the productivity of adult tea pickers with iron-fortified salt compared to no fortified salt. After one year, the average amount of tea picking was significantly higher in the intervention group than in the control group.

### 3.6. Results of Meta-Regression

The multivariable meta-regression analysis showed no significant association between the independent variables (hemoglobin concentrations before intervention, follow-up completeness, and length of follow-up) and the change in hemoglobin concentrations.

### 3.7. Risk of Bias Assessment

Only two trials provided enough information to classify random sequence generation and allocation concealment as adequate [18,26] (Table 3). The other 12 studies did not provide enough information to assess if procedures were performed adequately. Blinding of participants was mentioned in most of the trials. Seven studies addressed incomplete outcome data and 10 trials showed no selective outcome reporting (for example besides serum markers, also anemia, height/weight were reported).

In summary, the risk of bias for the investigated outcomes “hemoglobin change” and “anemia rates” is unclear in most of the studies and should be considered in the final conclusions.

**Table 3:** Risk of bias summary table of all 14 included RCT. Assessment categories: YES: criterion fulfilled; NO: criterion not fulfilled; “?”: unclear, not enough information given.

Reference	Author	Year	Adequate Sequence Generation?	Allocation Concealment?	Blinding?	Incomplete Outcome Data Addressed?	Are typical Outcomes Reported? (No Selective Outcome Reporting)
[25]	Andersson	2008	?	?	YES	YES	YES
[21]	Asibey-Berko	2007	?	YES	YES	NO	YES
[20]	Ballot	1989	?	?	YES	NO	NO
[33]	Chen	2005	?	?	YES	YES	YES
[32]	Huo	2002	?	?	?	NO	YES
[18]	Le	2007	YES	YES	YES	YES	YES
[15]	Longfis	2008	?	?	YES	YES	YES
[26]	Rajagopalan	2000	YES	YES	YES	NO	YES
[16]	Thuy	2005	?	?	YES	NO	YES
[17]	Thuy	2003	?	?	YES	YES	YES
[27]	Vinodkumar	2007	?	?	YES	YES	NO
[28]	Vinodkumar	2009	?	?	YES	YES	YES
[30]	Winichagoon	2006	YES	?	?	YES	YES
[24]	Zimmermann	2004	?	?	YES	YES	YES

## 4. Discussion

In our review with meta-analysis we studied hemoglobin, anemia, and functional outcomes of MN fortified condiments and noodles for children and adults. In summary, MN fortification (single MN strategy with iron or dual/multiple MN strategy with iron) showed a relevant increase in hemoglobin concentrations and decrease in anemia rates mainly for condiments. In our data set of 14 RCT, functional outcomes were rarely assessed (for example, morbidity, cognition, and productivity outcomes) and studies showed mixed results. Methodologic shortcomings of the primary studies were common and should be considered in the final conclusions. For example, details of randomization procedure (generation of random sequence; allocation concealment) were often not reported and it thus remains unclear if randomization was performed according to recommended standards. In addition, adherence to intervention was often not reported.

### 4.1. Existing Systematic Reviews and Research Needs

Other systematic reviews (SR) have evaluated the health effects of MN interventions. These SRs [5,9,36,37,38,39] differ from our review, either regarding the carrier (no condiments or contemporaneous analysis with other carriers) or because of the included designs of the studies (such as non-RCTs). Four of these SRs investigated the impact of MN fortified food on hemoglobin concentrations or anemia prevalence [5,9,36,39]. They all found an increased hemoglobin concentration or reduced risk of anemia due to fortified food with either iron [5,9,39], vitamin A and iron [5], or multiple micronutrients [5,36]. The other two reviews included primary studies with data for outcomes beyond hemoglobin concentrations and anemia rates, for example goiter prevalence or birth weight [37,38].

In our study, no difference in the increase of hemoglobin concentrations between fortification strategies (single/dual/multiple MN strategy) was found. This is somewhat surprising as other studies, which included mostly children and adolescents, showed an increased effect of multiple MN fortification compared to single MN fortification [6,7]. Possible explanations for this disagreement might be differential iron carriers or different target populations, as we included age groups from 5 to 50 years.

Standards for methodological rigor of primary studies in the nutrition field are an important research need as primary studies are the evidence base for reviews. The challenges and limitations of this type of research are highlighted by the result of our risk of bias assessment which addresses internal validity. For example, future studies in this field should adhere to established reporting standards for randomization procedure to give reviewers a better understanding if a possible selection bias has led to an overestimation of effect. Concerning external validity, more information about the representativeness of study participants for the real life target population is needed. For example, little socio-economic data was provided to estimate how well the study participants matched the profile of the most needy population groups of the country. Affordability is of outstanding importance to judge any public health effect of commercially distributed fortified food.

### 4.2. Implications for Decision Makers

Fortification of food and in particular condiments can be delivered in different ways. For example iodine fortified salt is a best practice example of a mandatory regulation by government in selected countries [40]. Salt is a widely used and low cost carrier for fortification. Other options like commercially distributed fortified food are also already available in many markets, even in low-income countries. A limitation of this market access is that the very poor population may not be reached. Each country has its own culture, economy, and social characteristics which will affect the delivery and management of fortified food to their population [41]. Thus, it may not be helpful to develop a single distribution strategy for all countries.

To better understand the impact of fortified food in “real world” settings, the monitoring of health effects is important. The Working Group [42] used a reasonable follow-up of 18 months for their large-scale effectiveness trial conducted in the general population of three rural areas and one urban area in India. The hemoglobin concentrations and anemia rates improved significantly in the intervention communities of this field trial after iron-fortification of salt. The change in Hb after 12 months ranged between 0.25g/dL and 3.28g/dL according to different areas, sex, and age. The prevalence of anemia before the intervention ranged between 6.5% and 99.2% and after the intervention between 6.5% and 60.9% according to different areas, sex, and age. Furthermore, in a recent review about food fortification in India observational studies provide evidence of positive health effects of salt fortification programs in real world settings [43].

Additional economic analyses should be performed to better understand the health economic effects of fortified condiments and noodles and support decision makers in their policy.

### 4.3. Strengths and Limitations

A strength of this review is the systematic approach adhering to critical methodological issues for synthesis [12] and reporting of evidence [13]. Even though we cannot be sure we have found all relevant studies, we are convinced that we found enough relevant studies to provide reliable data in this review.

There are also several limitations to be mentioned. First, the risk of bias in the included studies is unclear, due to poor reporting of the randomization procedure in the primary studies. Second, the statistical heterogeneity between the analyzed trials was high, thus pooled estimates have to be interpreted carefully. Third, we did not screen all references independently by two reviewers and did not perform independent, double data extraction or assessment of risk of bias. This could have led to bias. Fourth, we could have missed some studies because of the broad field of possible search terms for “condiments”. Finally, as only one noodle study was retrieved, there is insufficient aggregated evidence to conclude whether noodles by themselves improve health outcomes.

## 5. Conclusions

Micronutrient fortified condiments can be a strategy to reduce anemia in children and adults due to micronutrient deficiencies beyond supplementation programs and fortification of staple food. The effect of fortified noodles seems to be smaller, but conclusions are based on one study only. Risk of bias in the included studies is unclear and should be considered in the final conclusion.

## Supplementary Materials

The following are available online at http://www.mdpi.com/2072-6643/8/2/88/s1, Figure S1: Effect of fortified condiments and noodles on ferritin levels compared to non-fortified condiments or noodles. Included are 3 studies with suitable ferritin data for meta-analysis. Results are provided as weighted mean difference in ferritin (WMD: micro-g/L with 95%-CI) between intervention and control group.

## References

[1] World Health Organisation (2009). Global Health Risks: Mortality and Burden of Disease Attributable to Selected Major Risks.

[2] Allen L., de Benoist B., Dary O., Hurrell R. (2006). Guidelines on Food Fortification with Micronutrients.

[3] Bhutta Z.A., Ahmed T., Black R.E., Cousens S., Dewey K., Giugliani E., Haider B.A., Kirkwood B., Morris S.S., Sachdev H.P. (2008). What works? Interventions for maternal and child undernutrition and survival. Lancet.

[4] World Health Organization (2007). Conclusions and recommendations of the WHO consultation on prevention and control of iron deficiency in infants and young children in malaria-endemic areas. Food Nutr. Bull..

[5] Das J.K., Salam R., Kumar R., Bhutta Z.A. (2013). Micronutrient fortification of food and its impact on woman and child health: A systematic review. BioMed Central.

[6] Eichler K., Wieser S., Ruthemann I., Brugger U. (2012). Effects of micronutrient fortified milk and cereal food for infants and children: A systematic review. BMC Public Health.

[7] Allen L.H., Peerson J.M., Olney D.K. (2009). Provision of multiple rather than two or fewer micronutrients more effectively improves growth and other outcomes in micronutrient-deficient children and adults. J. Nutr..

[8] De-Regil L.M., Suchdev P.S., Vist G.E., Walleser S., Pena-Rosas J.P. (2011). Home fortification of foods with multiple micronutrient powders for health and nutrition in children under two years of age. Cochrane Database Syst. Rev..

[9] Gera T., Sachdev H.S., Boy E. (2012). Effect of iron-fortified foods on hematologic and biological outcomes: systematic review of randomized controlled trials. Am. J. Clin. Nutr..

[10] Haider B.A., Bhutta Z.A. (2011). Neonatal vitamin A supplementation for the prevention of mortality and morbidity in term neonates in developing countries. Cochrane Database Syst. Rev..

[11] Hess S.Y., Brown K.H. (2009). Impact of zinc fortification on zinc nutrition. Food Nutr. Bull..

[12] NHS Centre for Reviews and Dissemination (2008). CRD’s Guidance for Undertaking Reviews in Health Care.

[13] Moher D., Liberati A., Tetzlaff J., Altman D.G. (2009). Preferred reporting items for systematic reviews and meta-analyses: The PRISMA statement. Ann. Intern Med..

[14] Higgins J.P.T., Thompson S.G., Deeks J.J., Altman D.G. (2003). Measuring inconsistency in meta-analyses. BMJ.

[15] Longfils P., Monchy D., Weinheimer H., Chavasit V., Nakanishi Y., Schumann K. (2008). A comparative intervention trial on fish sauce fortified with NaFe-EDTA and FeSO_4_^+^ citrate in iron deficiency anemic school children in Kampot, Cambodia. Asia Pac. J. Clin. Nutr..

[16] Thuy P.V., Berger J., Nakanishi Y., Khan N.C., Lynch S., Dixon P. (2005). The Use of NaFeEDTA-Fortified Fish Sauce Is an Effective Tool for Controlling Iron Deficiency in Women of Childbearing Age in Rural Vietnam. J. Nutr..

[17] Thuy P.V., Berger J., Davidsson L., Khan N.C., Lam N.T., Cook J.D., Hurrell R.F., Khoi H.H. (2003). Regular consumption of NaFeEDTA-fortified fish sauce improves iron status and reduces the prevalence of anemia in anemic Vietnamese women. Am. J. Clin. Nutr..

[18] Le H.T., Brouwer I.D., Nguyen K.C., Burema J., Kok F.J. (2007). The effect of iron fortification and de-worming on anaemia and iron status of Vietnamese schoolchildren. Br. J. Nutr..

[19] Thi Le H., Brouwer I.D., Burema J., Nguyen K.C., Kok F.J. (2006). Efficacy of iron fortification compared to iron supplementation among Vietnamese schoolchildren. Nutr. J..

[20] Ballot D.E., MacPhail A.P., Bothwell T.H., Gillooly M., Mayet F.G. (1989). Fortification of curry powder with NaFe(111)EDTA in an iron-deficient population: Report of a controlled iron-fortification trial. Am. J. Clin. Nutr..

[21] Asibey-Berko E., Zlotkin S.H., Yeung G.S., Nti-Nimako W., Ahunu B., Kyei-Faried S., Johnston J.L., Tondeur M.C., Mannar V. (2007). Dual fortification of salt with iron and iodine in women and children in rural Ghana. East Afr. Med. J..

[22] Zimmermann M.B., Zeder C., Chaouki N., Saad A., Torresani T., Hurrell R.F. (2003). Dual fortification of salt with iodine and microencapsulated iron: A randomized, double-blind, controlled trial in Moroccan schoolchildren. Am. J. Clin. Nutr..

[23] Zimmermann M.B., Zeder C., Chaouki N., Torresani T., Saad A., Hurrell R.F. (2002). Addition of microencapsulated iron to iodized salt improves the efficacy of iodine in goitrous, iron-deficient children: A randomized, double-blind, controlled trial. Eur. J. Endocrinol..

[24] Zimmermann M.B., Wegmueller R., Zeder C., Chaouki N., Rohner F., Saissi M., Torresani T., Hurrell R.F. (2004). Dual fortification of salt with iodine and micronized ferric pyrophosphate: A randomized, double-blind, controlled trial. Am. J. Clin. Nutr..

[25] Andersson M., Thankachan P., Muthayya S., Goud R.B., Kurpad A.V., Hurrell R.F., Zimmermann M.B. (2008). Dual fortification of salt with iodine and iron: A randomized, double-blind, controlled trial of micronized ferric pyrophosphate and encapsulated ferrous fumarate in southern India. Am. J. Clin. Nutr..

[26] Rajagopalan S., Vinodkumar M. (2000). Effects of salt fortified with iron and iodine on the hamoglobin levels and productivity of tea pickers. Food Nutr. Bull..

[27] Vinodkumar M., Rajagopalan S., Bhagwat I.P., Singh S., Parmar B.S., Mishra O.P., Upadhyay S.S., Bhalia N.B., Deshpande S.R. (2007). A multicenter community study on the efficacy of double-fortified salt. Food Nutr. Bull..

[28] Vinodkumar M., Erhardt J.G., Rajagopalan S. (2009). Impact of a multiple-micronutrient fortified salt on the nutritional status and memory of schoolchildren. Int. J. Vitam. Nutr. Res..

[29] Manger M.S., McKenzie J.E., Winichagoon P., Gray A., Chavasit V., Pongcharoen T., Gowachirapant S., Ryan B., Wasantwisut E., Gibson R.S. (2008). A micronutrient-fortified seasoning powder reduces morbidity and improves short-term cognitive function, but has no effect on anthropometric measures in primary school children in northeast Thailand: A randomized controlled trial. Am. J. Clin. Nutr..

[30] Winichagoon P., McKenzie J.E., Chavasit V., Pongcharoen T., Gowachirapant S., Boonpraderm A., Manger M.S., Bailey K.B., Wasantwisut E., Gibson R.S. (2006). A multimicronutrient-fortified seasoning powder enhances the hemoglobin, zinc, and iodine status of primary school children in North East Thailand: A randomized controlled trial of efficacy. J. Nutr..

[31] Huo J., Sun J., Miao H., Yu B. (2001). Effect of NaFeEDTA fortified soy sauce on iron deficiency anemia in students. Wei Sheng Yan Jiu.

[32] Huo J., Sun J., Miao H., Yu B., Yang T., Liu Z., Lu C., Chen J., Zhang D., Ma Y. (2002). Therapeutic effects of NaFeEDTA-fortified soy sauce in anaemic children in China. Asia Pac. J. Clin. Nutr..

[33] Chen J., Zhao X., Zhang X., Yin S., Piao J., Huo J., Yu B., Qu N., Lu Q., Wang S. (2005). Studies on the effectiveness of NaFeEDTA-fortified soy sauce in controlling iron deficiency: A population-based intervention trial. Food Nutr. Bull..

[34] Lee Y.M., Skurk T., Hennig M., Hauner H. (2007). Effect of a milk drink supplemented with whey peptides on blood pressure in patients with mild hypertension. Eur. J. Nutr..

[35] World Health Organization (2011). Haemoglobin concentrations for the diagnosis of anaemia and assessment of severity. Vitamin and Mineral Nutrition Information System.

[36] Best C., Neufingerl N., Del Rosso J.M., Transler C., van den Briel T., Osendarp S. (2011). Can multi-micronutrient food fortification improve the micronutrient status, growth, health, and cognition of schoolchildren?. A systematic review. Nutr. Rev..

[37] Jiang T., Xue Q. (2010). Fortified salt for preventing iodine deficiency disorders: A systematic review Chin. J. Evid. Based Med..

[38] Vucic V., Berti C., Vollhardt C., Fekete K., Cetin I., Koletzko B., Gurinovic M., van't Veer P. (2013). Effect of iron intervention on growth during gestation, infancy, childhood, and adolescence: A systematic review with meta-analysis. Nutr. Rev..

[39] Casgrain A., Collings R., Harvey L.J., Hooper L., Fairweather-Tait S.J. (2012). Effect of iron intake on iron status: A systematic review and meta-analysis of randomized controlled trials Am. J. Clin. Nutr..

[40] Van den Wijngaart A., Begin F., Codling K., Randall P., Johnson Q.W. (2013). Regulatory monitoring systems of fortified salt and wheat flour in selected ASEAN countries. Food Nutr. Bull..

[41] Pena-Rosas J.P., De-Regil L.M., Rogers L.M., Bopardikar A., Panisset U. (2012). Translating research into action: WHO evidence-informed guidelines for safe and effective micronutrient interventions. J. Nutr..

[42] The Working Group (1982). Use of common salt fortified with iron in the control and prevention of anemia—A collaborative study. Am. J. Clin. Nutr..

[43] Liu P., Bhatia R., Pachon H. (2014). Food fortification in India. Indian J. Community Health.

